# Osteoporosis Can Be the Sole Presentation in Celiac Disease

**DOI:** 10.7759/cureus.20602

**Published:** 2021-12-22

**Authors:** Veera Jayasree Latha Bommu, Lubna Mirza

**Affiliations:** 1 Internal Medicine, Hackensack Meridian Ocean Medical Center, Brick, USA; 2 Endocrinology, Norman Regional Hospital, Norman, USA

**Keywords:** elevated alkaline phosphatase, celiac sprue, osteopenia, gluten enteropathy, gluten intolerance, celiac disease, osteoporosis

## Abstract

Celiac disease, an autoimmune condition causing gluten intolerance and disrupted absorption of nutrients, predisposes to osteoporosis. The release of pro-inflammatory cytokines, calcium malabsorption, and the activation of osteoclasts represent the main mechanisms responsible for bone derangement. This is evidenced by the low T-score on dual-energy x-ray absorptiometry (DXA) scans in these patients. However, these changes are reversible with the early initiation of a gluten-free diet. Hence, it is important for physicians to consider screening for celiac disease panel in patients presenting with osteoporotic features with no clear etiology.

## Introduction

Celiac disease, otherwise known as “celiac sprue,” is an autoimmune disorder of the small intestine caused by the ingestion of gluten products in susceptible people. Celiac disease is not uncommon, as believed originally, with a 1% global prevalence. In celiac patients, gluten ingestion causes enteropathy because of the disruption of the mucosal surface and therefore leads to decreased absorption of nutrients. The pathogenesis of non-celiac gluten sensitivity (NCGS) includes multiple factors but remains largely unknown [1]. This is a chronic condition that needs lifetime avoidance of gluten. Celiac disease patients can present with many gastrointestinal symptoms like diarrhea, flatulence, and fatty stools. Half of them are asymptomatic or present with atypical symptoms such as anemia, bone issues, weight loss, and skin rash. However, osteoporosis is the sole and unique presentation of celiac disease. Here, we are reporting a case of osteoporosis in a 63-year-old male patient, which leads to the diagnosis of celiac disease as the underlying pathology.

## Case presentation

A 63-year-old male patient was referred to our endocrinology clinic for elevated serum alkaline phosphatase levels (ALP) of 143 IU/L (25-100 IU/L) and elevated intact parathyroid hormone (PTH) of 149.5 pg/ml (10-65 pg/ml) but his vitamin D3 was normal (Table 1). His medical history included arthritis, back pain, hypercholesterolemia, essential hypertension, and chronic obstructive pulmonary disease (COPD). His surgical history included cholecystectomy, decompressive surgery for carpal tunnel syndrome, and polypectomy for colon polyp that was incidentally found on colonoscopy. His medications included vitamin D3 and B12 supplements in addition to daily aspirin, tamsulosin, losartan, celecoxib, montelukast, hydrochlorothiazide, atorvastatin, pantoprazole, and prednisone. Family history was significant for hyperlipidemia. He was a former smoker and quit 20 years back but drank alcohol occasionally. His vital signs were normal and physical examination revealed no abnormalities. On reviewing his blood work further, his serum bone alkaline phosphatase was elevated at 60.2 IU/L while other isoenzymes were normal and serum calcium was low at 8 mg/dl (8.4-10.2 mg/dl). All the other laboratory tests including comprehensive metabolic panel (CMP), thyroid-stimulating hormone (TSH), glycated hemoglobin (HbA1c), vitamin B12, and iron profile, were normal except hemoglobin was 13.1g/dl (13.5-17.5 g/dl) and hematocrit was 39.8% (41%-53%). Therefore, Paget’s disease was suspected, and we advised him to take 1200 mg of calcium supplements daily and ordered 24-hour urinary calcium, ionized calcium, phosphorous, and a dual-energy X-ray absorptiometry (DEXA) scan.

**Table 1:** Lab values on the initial visit vs 3 months later N/A: Not available

	Initial Visit	3 months later
Alkaline phosphatase	143 IU/L(H)	145 IU/L(H)
Intact PTH	149.5 pg/ml (H)	151 pg/ml(H)
25 (OH) Vitamin D	64pg/ml(N)	72pg/ml(N)
Ionized Calcium	N/A	1.2 mmol/L
24-hour urinary calcium	N/A	<16mg/24hrs

On follow-up 3 months later, his alkaline phosphatase, intact parathyroid hormone levels were still elevated and elevated 1, 25-dihydroxy vitamin D at 72 pg/ml, and ionized calcium was normal at 1.2 mmol/l while 24-hour urinary calcium was very low at less than 16 mg in 24 hours (Table 1). His dual X-ray absorptiometry (DXA) bone density test revealed osteopenia at the worst T-score of -2.4 in the right femoral neck. So, we screened him with Androgen Deficiency in the Aging Male's (ADAM's) questionnaire, and he had all 10 questions positive. On further questioning, he stated that he had decreased libido. Considering the low bone density score, the differential diagnoses to consider included pseudo-hypoparathyroidism, Paget’s disease, familial hypocalciuric hypercalcemia, and male hypogonadism. So, nuclear bone scans and labs for low testosterone workup were ordered in addition to genetic testing for calcium-sensing receptor mutation (Invitae, California).

On review later, his nuclear bone scan results showed areas of mildly increased uptake in a few joints suggesting degenerative joint disease. However, there were no foci of Paget’s disease. Genetic testing for calcium-sensing receptors revealed no abnormalities. The celiac disease panel showed elevated serum immunoglobulin A (IgA) at 445 mg/dl, elevated tissue transglutaminase antibodies IgA at >100 U/ml, which raised a high suspicion of celiac disease. Therefore, we referred the patient to a gastroenterologist for biopsy and a dietitian for nutrition education. He was advised to follow a gluten-free diet and was prescribed alendronate 70 mg for six months.

## Discussion

Celiac disease is caused due to genetic and environmental factors. The environmental trigger constitutes gluten, whereas the genetic predisposition has been recognized in the major histocompatibility complex region. The importance of gluten in the pathology of celiac disease was discovered in 1953, whereas celiac disease was first detected in 1888. Celiac disease causes hypersensitivity to gluten [2]. Celiac disease is substantially more common in osteoporotic people (3.4%) than in non-osteoporotic people (0.2%) [3]. Celiac disease is associated with a number of other autoimmune disorders, including autoimmune thyroid disease and type 1 diabetes mellitus. One particular association was established with chromosome 15q26, which has a type 1 diabetes susceptibility locus, and with chromosome 5q and possibly 11q [2].

The pathophysiology of celiac disease is known to be an increased number of intraepithelial lymphocytes, which bear infrequent gamma-delta T-cell receptors in patients with active, gluten-sensitive celiac sprue when compared to normal subjects. Moreover, patients with refractory sprue have aberrant lymphocytes as well but with restricted gene rearrangements. The intraepithelial T lymphocytes show increased expression of interferon-gamma and IL-10 [4-5]. Many recent studies have suggested the role of local and systemic inflammation in the development of osteopenia and osteoporosis in celiac disease [6-7]. Tumor necrosis factor-alpha (TNFα) and interleukin-1 (IL-1) cause bone resorption. IL-6 recruits osteoclasts and promotes their differentiation. These can have a direct negative effect on bone density, which is independent of vitamin D levels and PTH levels. The second theory of osteoporosis is also due to lack of vitamin D and calcium absorption, which leads to secondary hyperparathyroidism. Patients with celiac disease have lower vitamin D levels and higher PTH levels on presentation [8]. The increased bone resorption raises alkaline phosphatase levels, which explains the presentation of this patient.

Classically, patients with celiac disease present with diarrhea, weight loss, and other gastrointestinal (GI) symptoms. However, more than half of the patients with celiac disease do not show classic presentation, and some are even asymptomatic; they rather present as asymptomatic or with nonspecific features of nutritional deficiency. Non-celiac gluten sensitivity (NCGS), a new entity that is not driven by immune response has been included. Low bone mineral density is one of the atypical presentations of celiac disease.

The signs and symptoms of celiac disease include GI symptoms, such as diarrhea, flatulence, borborygmus, weight loss, weakness, and fatigue, usually related to general poor nutrition and severe abdominal pain, and in infants and young children can present with failure to thrive and growth retardation if left untreated. Extra-intestinal symptoms may include anemia, osteopenia, and osteoporosis. Skin involvement leads to dermatitis herpetiformis, a condition with papulovesicular, pruritic skin lesions over the extensor surfaces of the extremities, buttocks, trunk, neck, and scalp. Neurologic symptoms include paresthesia with sensory loss, motor weakness, ataxia, and seizures. A bleeding diathesis due to prothrombin deficiency as a result of impaired absorption of fat-soluble vitamin K. Hormonal disorders include delayed menarche, amenorrhea, infertility in women, and impotence and infertility in men as a result of impaired absorption of vitamin E [3]. Undiagnosed celiac disease might result in severe complications in adults as well as in children.

The American College of Gastroenterology (ACG) recommends that testing for immunoglobulin-A anti-tissue transglutaminase antibody (IgA TTG) is the best test for suspected celiac disease despite the fact that biopsies are needed for confirmation. An IgA TTG test along with the testing of IgG-deamidated gliadin peptides should be considered in children younger than two years [9]. Other laboratory tests include regular monitoring of fat-soluble vitamin deficiencies, serum IgA antibodies, and electrolytes for any malnutrition. Imaging studies, such as barium meal X-ray, are helpful to diagnose untreated celiac disease, in which dilatation of the small intestine, a coarsening or obliteration of the normally delicate mucosal pattern, and fragmentation or flocculation of the barium in the gut lumen can be visualized [10].

The primary treatment of celiac disease is avoidance of gluten in the diet. It includes abstinence from wheat, barley, rye, green spelt, spelt, and all ready-made products, such as noodles, which are manufactured from the above [11]. However, complete abstinence of gluten-containing grain products, such as wheat flour, which is virtually ubiquitous in the American diet, is somewhat difficult. A small percentage of celiac disease patients may not respond to a gluten-free diet. In these patients who are refractory to dietary modification, corticosteroids may be helpful [12].

This patient presented with raised alkaline phosphatase levels and osteopenia. Unlike many celiac disease patients, his 25-hydroxy vitamin D levels and PTH levels were high at 149.5 as shown in Table 1. After an extensive workup that ruled out many possible causes of such a presentation, a celiac disease panel was performed, which came back positive. Normal vitamin D levels and elevated PTH levels do not explain the pathophysiology hypothesized for low bone density in celiac disease and suggest other underlying factors to this phenomenon. Our patient was then referred to a gastroenterologist and a dietitian. He was advised to comply with a gluten-free diet for his lifetime and was prescribed alendronate 70 mg for the next six months for his osteoporosis.

## Conclusions

The classification of celiac disease as a gastrointestinal disease with gastrointestinal manifestations leads to underdiagnosing or misdiagnosing asymptomatic or atypically presenting patients. Our case report recommends that a celiac disease panel should be considered in all osteoporotic and osteopenic patients. Since the diagnosis and subsequent management with a gluten-free diet are essential in preventing disease progression, it is important to recognize celiac disease as a potential culprit in patients with low bone density.
